# Dietary Effects of Four Phytoecdysteroids on Growth and Development of the Indian Meal Moth, *Plodia interpunctella*

**DOI:** 10.1673/031.010.1301

**Published:** 2010-03-02

**Authors:** Kacem Rharrabe, Fouad Sayan, René LaFont

**Affiliations:** ^1^ PER - Centre des Etudes Environnementales Méditerranéennes, Laboratoire de Biologie Appliquée & Sciences de l'Environnement, Université Abdelmalek Essaadi, Faculté des Sciences et Techniques, BP 416, Tangier, Morocco; ^2^ Université Pierre et Marie Curie, Laboratoire BIOSIPE (ER3) Quai St. Bernard, 75252 Paris 05, France

**Keywords:** *Ajuga iva*, *Silene nutans*, bioinsecticides, Phytoecdysteroids, phytophagous insect, toxicity

## Abstract

Using pure phytoecdysteroids isolated from *Ajuga iva* (L.) Schreber (Lamiales: Lamiaceae) and *Silene nutans* L. (Caryophyllales: Caryophyllaceae), plants known for their high ecdysteroid content, a study was carried out on the effects of ingestion of four different phytoecdysteroids (20-hydroxyecdysone, polypodine B, ponasterone A and makisterone A) on the growth and development of the Indian meal moth, *Plodia interpunctella* Hübner (Lepidoptera: Pyralidae) larvae when added at a concentration of 200 ppm in their diet. The experiments clearly showed the susceptibility of *P. interpunctella* to phytoecdysteroid ingestion. The toxicity of phytoecdysteroids manifested itself by a decrease in larval weight, induction of cannibalism and an increase of mortality, together with disruption of development. The severity of the phytoecdysteroid effect on *P. interpunctella* depended on the structure of the molecule. The results demonstrate that the minimal structural differences existing between these four phytoecdysteroids significantly affected their toxicity toward *P. interpunctella.* Makisterone A was the most toxic of the four compounds towards *P. interpunctella* larvae. In conclusion, phytoecdysteroids ingestion evokes disruptive growth effects on *P. interpunctella.* This work supports a role for phytoecdysteroids in plant defence against phytophagous insects.

## Introduction

The study of insect-plant interactions is currently one of the most actively investigated areas in chemical ecology, partly owing to its interesting perspectives for the development of new biopesticides with plant origins. These interactions involve numerous secondary plant metabolites that may interfere with behaviour, growth and/or development of insects. Phytoecdysteroids are secondary metabolites produced by many plants. They represent analogues of insect steroid hormones (ecdysteroids) that control insect growth, development, and reproduction (Koolman 1989; Lafont 1997). It has been suggested that they form part of the plant's defences against phytophagous insects (Lafont 1997; Adler and Grebenok 1999) and soil nematodes (Soriano et al. 2004).

Several studies have investigated the effects of phytoecdysteroids on phytophagous insects. The exogenous application of phytoecdysteroids to a number of species resulted in marked growth and developmental disruption, e.g. in *Spodoptera frugiperda* (Kubo et al. 1983), *Bombyx mort* (Tanaka and Takeda 1993a, 1993b), *Lobesia botrana* (Mondy et al. 1997), *Inachis io* and *Aglais urticae* (Blackford and Dinan 1997a), and *Bradysia impatiens* (Schmelz et al. 2002). This disruption involves a number of effects including inhibition of growth, induction of supernumerary larval instars, death without moulting, and death during or after induced moulting. However, certain insect species remain unaffected by dietary phytoecdysteroids. This is the case for *Heliothis virescens* (Kubo et al. 1987), *Heliothis armigera* (Robinson et al. 1987), *Spodoptera littoralis* (Blackford et al. 1996), and *Lacanobia oleracea* (Blackford and Dinan 1997b). These species have developed effective detoxification mechanisms against ingested phytoecdysteroids.

Due to the obvious differences in the susceptibility of lepidopterans to ingested phytoecdysteroids, it is of interest to determine whether other lepidopteran pests, such as *Plodia interpunctella* Hübner (Lepidoptera: Pyralidae), can tolerate ingested phytoecdysteroids and of interest to analyze their relative susceptibility to different molecules of this family.

The Indian meal moth, *P. interpunctella,* is a world-wide insect pest of stored-products and processed food commodities (Mohandass et al. 2007). In Morocco, this insect is a major problem during processing and storage of dried fruit such as dates (Azelmat et al. 2005).

In this work, the effects of four phytoecdysteroids on the development of *P. interpunctella* were studied. The phytoedysteroids used were 20-hydroxyecdysone (20E), polypodine B (PolB), ponasterone A (PonA), and makisterone A (MakA). These were used in preference to other phytoecdysteroids because they are among the most common phytoecdysteroids present in plants (Dinan 2001). In addition, the minimal structural differences between these molecules were assessed to determine their effects on toxicity toward *P. interpunctella.*

## Materials and Methods

### Insect rearing

*P. interpunctella* were collected as larvae infesting dates from the Errachidia province in the southeast region of Morocco. The larvae were reared under standard conditions at 28 ± 2° C with a relative humidity of 70 ± 5 % and a photoperiod of 16:8 L:D. Insects were placed in 0.25 L glass containers half-full of wheat flour as a medium. Emerging adults were removed and allowed to mate in new 0.25 L glass containers. Eggs were allowed to develop in their oviposition sites.

Under these conditions, *P. interpunctella* displays 5 larval instars and the life-cycle lasts 36 to 44 days.

### Phytoecdysteroid extraction and purification

**Figure 1:**
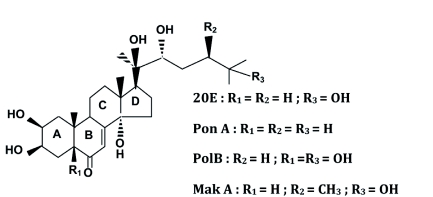
Structures of the phytoecdysteroids used in this study. 20E: 20-hydroxyecdysone, PolB: polypodine B, PonA: ponasterone A, MakA: makisterone A. High quality figures are available online.

Phytoecdysteroids were isolated from two plants known for their high phytoecdysteroid content. The phytoecdysteroids 20E and MakA were purified from *Ajuga iva* Schreber (Lamiales: Lamiaceae), and 20E and PolB were purified from *Silene nutans* L. (Caryophyllales: Caryophyllaceae). PonA was prepared chemically from 20E (Dinan 1985). The purity of all molecules was checked by HPLC and was superior to 95%. The structures of the 4 analogues are shown in Figure 1. Plants for extraction and purification of phytoecdysteroids were collected from different sites. *A. iva* was collected around the Tangier region (Morocco), and *S. nutans* was collected in the Pradelles region (Haute-Loire, France).

Classical procedures based on liquid-liquid partitions and different types of chromatography (low-pressure column chromatography and HPLC) were used for phytoecdysteroid extraction and purification (e.g. Báthori 1998).

### Treatments

Each phytoecdysteroid was dissolved in 5% (v/v) methanol in distilled water. A volume of 5 ml was incorporated into 5 g wheat flour at 200 ppm. For control larvae, 5 ml of 5% methanol in distilled water were added to wheat flour. The solvent was evaporated from the diet at 35° C in an oven over a period of 48 hours. To verify that the effect was due to phytoecdysteroid ingestion and not to a deterrency effect, another experimental group (starved) was generated by transferring larvae into Petri dishes with no food. The choice of a concentration of 200 ppm was based on previous results that have been obtained with different concentrations of 20E. In fact, 200 ppm 20E corresponded to its EC50 on *P. interpunctella* larvae. The use of other phytoecdysteroids was to compare their effects to those of 20E and possibly to identify more efficient molecules.

### Post-embryonic development

Fourth instar larvae were starved for 24 h prior to use to induce a higher feeding rate. Then, 10 larvae were placed in a Petri dish in the presence of either 5 g treated or 5 g control diet. Observations, including larval weight, mortality, cannibalism, pupation and adult emergence, were made every second day over 30 days. Mortality was identified by brown colouration with no observable movements. Cannibalism was noted by the disappearance of larval bodies from the Petri dishes. Larvae were maintained under standard conditions throughout the experiment. Five replicates were performed for treated, starved, and control larvae.

### Statistical analysis

To determine the statistical significance of treatment effect, results were analysed by ANOVA Statistica Software using the Tukey HSD test (Statistica 1997). A significance level of 0.05 was applied to all statistical tests.

## Results

### Weight

**Figure 2:**
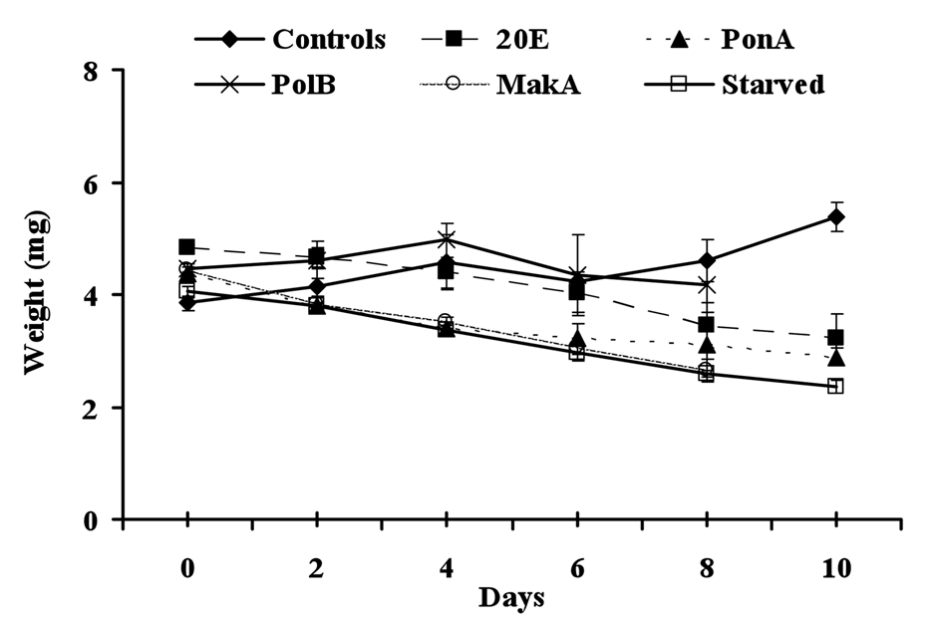
Effect of different phytoecdysteroids, at a concentration of 200 ppm, on *P. interpunctella* larval weight. Each data point represents the mean ± standard error of five replicates. High quality figures are available online.

The results of weight change in treated, starved, and control larvae are shown in Figure 2. Phytoecdysteroid ingestion had a negative effect on the evolution of larval weight. During the first days, a slight decrease of weight of treated insects was recorded for each compound, except for PolB, where there was a slight increase in weight. This effect increased with time. Thus, 10 days after treatment started, weight loss was 33.4%, 34.1%, and 40.3% for larvae fed 20E, PonA, and MakA, respectively. For larvae fed PolB, the weight increased during the first four days before registering a decrease from the sixth day forward. Overall, the weight loss was only 6.3%. For starved larvae, the weight reduction was more pronounced (43%). In contrast, control larvae gained 28.6% in weight. Statistical analysis showed that the treatment with 20E, PonA, or MakA, as well as starvation, had a very highly significant, negative effect on larval weight evolution (p < 0.001). For PolB, the effect was not significant when compared with control larvae. The effect of starvation was significantly more pronounced when compared between the 20E and PolB groups (p < 0.001).

### Mortality

**Figure 3:**
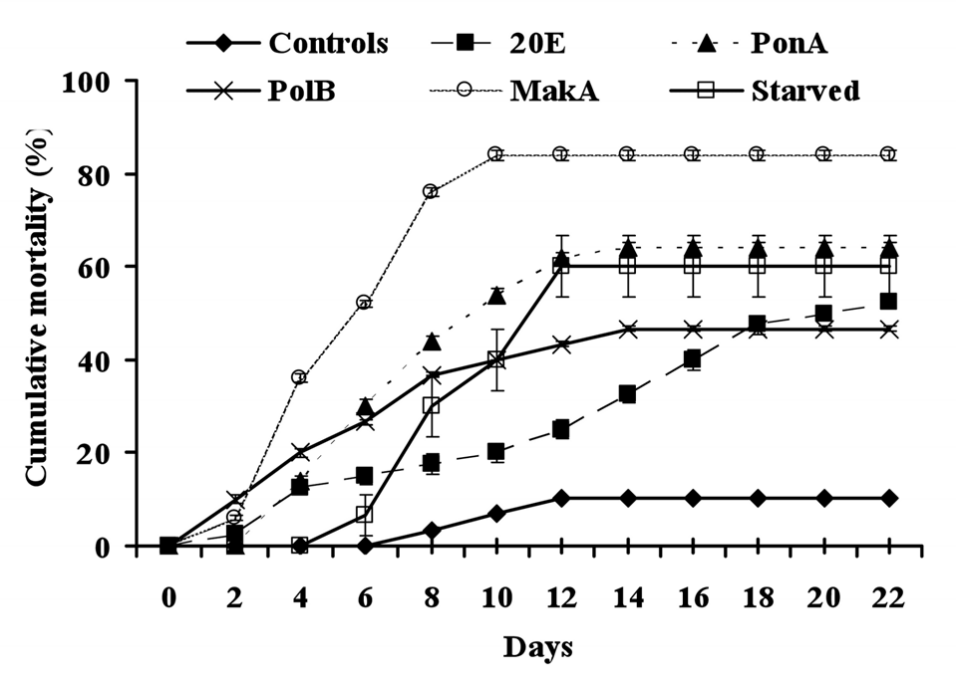
Effect of different phytoecdysteroids, at a concentration of 200 ppm, on *P. interpunctella* larval mortality. Each data point represents the mean ± standard error of five replicates. High quality figures are available online.

Phytoecdysteroid ingestion also caused high larval mortality (Figure 3). The mortality started two days after the beginning of treatment for 20E, MakA, and PolB, and from the fourth day for PonA. It increased throughout the observation period. After eight days, it was 17.5%, 36.7%, 44%, and 76% for 20E, PolB, PonA, and MakA, respectively. Then, 22 days after the beginning of the experiment, it was 46.7%, 52.5%, 64%, and 84% for PolB, 20E, PonA and MakA, respectively. In starved larvae, mortality appeared after six days of treatment, and it was 60%) on day twelve. For controls, the larval mortality did not exceed 11% during the whole experiment. ANOVA analysis showed that for all treatments and for starvation there was a very highly significant larval mortality as compared with controls (p < 0.001). For starvation compared to treated groups, the significant difference was noted compared to MakA and PolB (p < 0.001).

### Cannibalism

**Figure 4:**
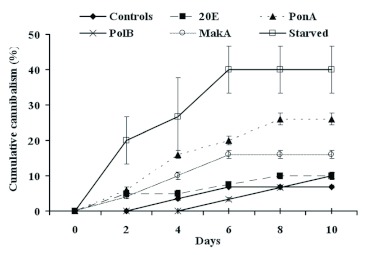
Effect of different phytoecdysteroids, at a concentration of 200 ppm, on *P. interpunctella* larval cannibalism. Each data point represents the mean ± standard error of five replicates. High quality figures are available online.

The presence of phytoecdysteroids in the larval diet provoked high levels of cannibalistic behaviour between larvae (Figure 4). We noted the disappearance of larval bodies in the batches treated with 20E, PonA and MakA from the second day, with a rate that varied between 4 and 6%. This percentage increased with time. It reached 10%, 16% and 26% for 20E, MakA and PonA, respectively. For PolB, cannibalism appeared only six days after the treatment began and it reached its maximum on day ten at 10%. For starved larvae, the cannibalism was more pronounced, being 40% after just six days. The rate of cannibalism in control larvae did not surpass 7%. Statistical analysis showed that the treatment with PonA, as well as starvation, had a very highly significant effect on cannibalism (P < 0.001) and a highly significant effect was observed for MakA (P < 0.01). The effect of starvation was highly more pronounced across with all phytoecdysteroid treatments (P < 0.001).

**Figure 5:**
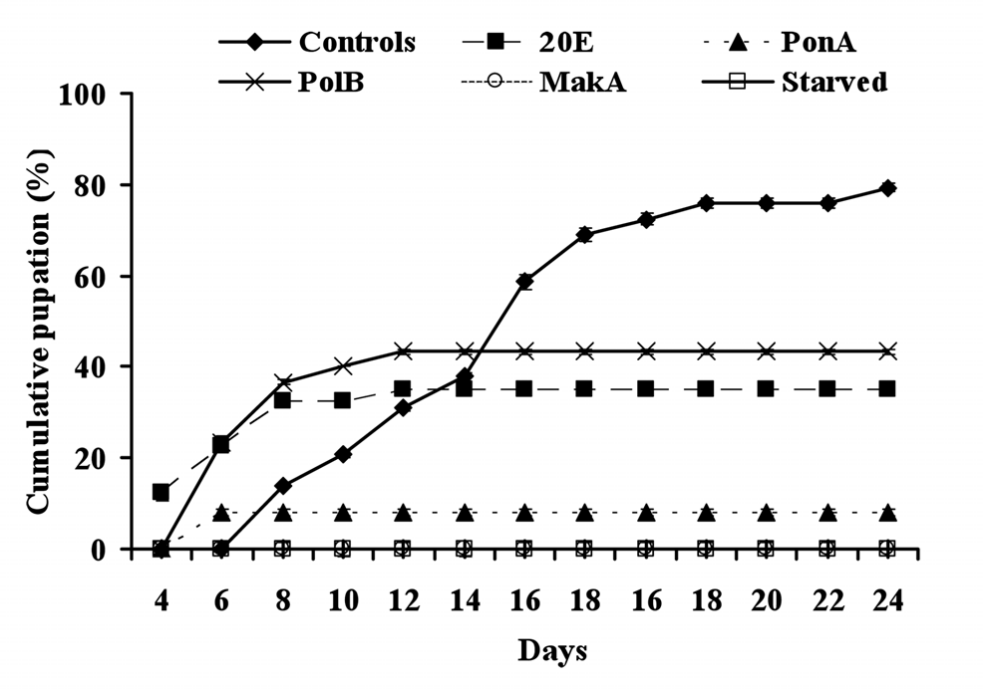
Effect of different phytoecdysteroids, at a concentration of 200 ppm, on *P. interpunctella* pupation. Each data point represents the mean ± standard error of five replicates. High quality figures are available online.

### Pupation

It seems clear that pupation was affected by phytoecdysteroid ingestion (Figure 5). In larvae fed MakA, pupation was completely inhibited, as in starved insects (p < 0.001). However, for other molecules, precocious pupation appeared by two days after for larvae fed PonA or PolB and four days in the case of 20E as compared with control group. The maximum pupation percentage was 43.4%, 35%, and 8% for PolB, 20E, and PonA, respectively (p < 0.001). Pupation of control larvae began on day 8 and reached 79% over 28 d.

### Adult emergence

**Figure 6:**
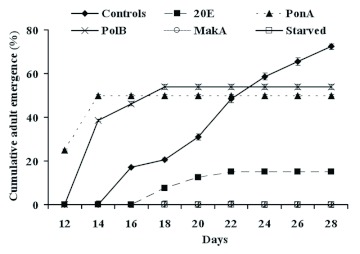
Effect of different phytoecdysteroids, at a concentration of 200 ppm, on *P. interpunctella* adult emergence. Each data point represents the mean ± standard error of five replicates. High quality figures are available online.

From the pupae, the percentages of adults emerging were very different between experimentally treated larvae (Figure 6). Moreover, as for pupation, this developmental parameter was significantly affected by the presence of phytoecdysteroids in the larval diet (p < 0.001). For insects fed phytoecdysteroids, an acceleration of adult emergence by two and four days for PonA and PolB, respectively, was noted when compared with controls. In contrast, in the case of 20E, a delay of two days was recorded. At the end of the experiment (after 30 days), the percentage of adult emergence was 15%, 50%, and 54% for 20E, PonA, and PolB, respectively. For untreated larvae, it occurred over sixteen days and reached 72% by day 28.

## Discussion

A number of studies have investigated insect susceptibility to dietary phytoecdysteroids. Most of the species examined have been lepidoptera, due to the pre-eminence of this order among phytophagous pests. Some species are very tolerant, such as *H. virescens* (Kubo et al. 1987), *H. armigera* (Robinson et al. 1987), and *S. littoralis* (Blackford et al. 1996). Other species, *Cynthia cardui* and *Tyria jacobaeae,* for example, are semitolerant (Blackford and Dinan 1997a), while others are highly susceptible, such as *S. frugiperda, Pectinophora gossypiella* (Kubo et al. 1981, 1983), *Acrolepiopsis assectella* (Arnault and Sláma 1986), *A. urticae,* and *I.* *io* (Blackford and Dinan 1997a).

The data presented in this report clearly show the susceptibility of *P. interpunctella* to phytoecdysteroid ingestion. The toxicity of phytoecdysteroids is associated with a decrease in larval weight, induced cannibalism, increased mortality, together with disruption of development. Such effects have been observed in *P. interpunctella* reared on other pure allelochemicals with insecticidal activity, such as harmaline (Rharrabe et al. 2007a) and azadirachtine (Rharrabe et al. 2008). The addition of phytoecdysteroids to the diet of *P. interpunctella* resulted in an array of metamorphic disorders. These effects can be divided into three categories: (i) precocious pupation and adult emergence, (ii) delay in adult emergence (in the case of 20E), and (iii) reduction in pupation and adult emergence.

These larval growth and developmental defects could result from the cytotoxicity of phytoecdysteroids on the larvae's midgut. In fact, 20E ingestion caused, in a dose-dependent manner, a severe cytotoxicity of the midgut epithelial cells of *P. interpunctella* larvae (Rharrabe et al. 2009). Tanaka and Yukuhiro (1999) also found that, in *B. mori* larvae fed 20E, the morphology of midgut epithelial cells was disrupted.

Exposure of *P. interpunctella* larvae to phytoecdysteroids induced significant cannibalism. Cannibalistic behaviour is a common phenomenon in arthropods, including insects (Polis 1988). Cannibalism is commonly increased under stressful conditions, such as high population density or scarce food, when cannibals obtain a nutritional benefit (Via 1999). In *Helicoverpa zea* larvae reared on *Bacillus thuringiensis* transgenic corn, the cannibalism was enhanced, and this diet appeared to represent a particularly stressful environment for this -insect (Chilcutt 2006). For *P. interpunctella,* incorporation of harmaline into the larval diet provoked high levels of cannibalistic behaviour (Rharrabe et al. 2007a).

Starved *P. interpunctella* larvae displayed a marked disruption of development with total prevention of pupation, extensive cannibalism, and high mortality. However, the intensity of these perturbations differed from those caused by phytoecdysteroid ingestion. For example, the weight loss was more pronounced in starved larvae than in larvae fed phytoecdysteroids. On the other hand, mortality in starved larvae began four days later than in larvae treated with phytoecdysteroids, and it reached a maximum just after six days.

The effects of phytoecdysteroids on *P. interpunctella* depend on the analogue used and the developmental parameters observed. These results show that even the small structural differences between these four phytoecdysteroids significantly affect their toxicity toward *P. interpunctella.* Similar observations were recorded in other species. For example, in *B. mori,* the fate of the larvae depended on the ecdysteroid analogue; the addition of ecdysone caused supernumerary moults (Tanaka and Takeda 1993a), while 20E provoked synchronised moults at low doses and death at high doses (Tanaka and Takeda 1993b). This difference in sensitivity to ingested phytoecdysteroids might have different explanations, for example: (i) differences of the target site(s) of each analogue in larvae, (ii) different rates of uptake of each analogue by larvae, or (iii) difference in the ecdysteroids detoxification rates/pathways. Insects have developed diverse detoxification mechanisms toward ingested ecdysteroids, and in *P. interpunctella* larvae, these include oxidation at C3 and conjugation with fatty acids of the 22-OH group (Rharrabe et al. 2007b). Among the tested molecules, MakA showed the highest toxicity toward *P. interpunctella* larvae. This could be explained by the extra methyl group in position C-24 that may make 22-acylation less efficient and therefore may increase its half-life. This explanation, however, requires direct experimental evidence.

These results in *P. interpunctella* (a polyphagous species) do not fully fit with the hypothesis of Blackford and Dinan (1997a), which proposed that the physiological and the behavioural sensitivity of insects to phytoecdysteroids is correlated to their feeding habits (monophagous = sensitive, oligophagous = semi-tolerant, and polyphagous = tolerant). The reported susceptibility of *P. interpunctella* may be correlated with the fact that it feeds on food commodities that are phytoecdysteroid-negative. At the other extreme, truly polyphagous species, such as *S. littoralis* and *L. oleracea,* feed on richly ecdysteroid-containing plants without exhibiting any abnormal effects, due to the presence of a very efficient detoxification pathway. These species have such a wide host-plant range that there is a very high probability that their diet will include phytoecdysteroids (in Blackford and Dinan 1997a).

In summary, this work establishes the potent growth inhibitory effects of phytoecdysteroids on *P. interpunctella.* They caused significant disturbance to growth and development, confirming that phytoecdysteroids can be a valuable plant defence against insect pests. It would be of interest to check the activity of some other phytoecdysteroids on *P. interpunctella* and to perform similar experiments on other species in order to determine which compounds are the most efficient in every case. This could provide a reason for the presence of complex ecdysteroid cocktails in plants (Bâthori et al. 1999).

Together, these results and the literature data indicate that phytoecdysteroids play a role as defensive substances against phytophagous insects. But, is it feasible to use phytoecdysteroids for crop protection against insect pests? To answer this question, much work is necessary in the laboratory and also in the field. For the moment, certain strategies could be foreseen. First, the use of such compounds by treating with the extracts of plants rich in phytoecdysteroids is difficult to conceive, except in confined spaces, like the conservation of stored-products in stock. Second, it is possible to imagine cultivated plants protecting themselves against insect pests by stimulating them to produce phytoecdysteroids. Indeed, the taxonomical distribution of plants producing ecdysteroids suggests that the genes necessary for their production are widespread in the plant kingdom (Dinan 2001).

The use of phytoecdysteroids to fight against insect pests is not an alternative for the methods used currently, but they represent interesting molecules that could have an important part in integrated pest management strategies for cultures and stored products.

## Abbreviation

20E: 20-hydroxyecdysone,
PolB: polypodine B,
PonA: ponasterone A,
MakA: makisterone A
